# Quantification of the energy gap in young overweight children. The PIAMA birth cohort study

**DOI:** 10.1186/1471-2458-11-326

**Published:** 2011-05-17

**Authors:** Saskia W van den Berg, Jolanda MA Boer, Salome Scholtens, Johan C de Jongste, Bert Brunekreef, Henriette A Smit, Alet H Wijga

**Affiliations:** 1 National Institute for Public Health and the Environment (RIVM), Bilthoven, the Netherlands; 2 Institute for Risk Assessment Sciences (IRAS), Division Environmental Epidemiology, University Utrecht, Utrecht, The Netherlands; 3 Department of Pediatrics/Respiratory Medicine, Erasmus Medical Center/Sophia Children's Hospital, Rotterdam, The Netherlands; 4 Julius Center for Health Sciences and Primary Care, University Medical Center Utrecht, Utrecht, The Netherlands

## Abstract

**Background:**

Overweight develops gradually as a result of a long term surplus on the balance between energy intake and energy expenditure. Aim of this study was to quantify the positive energy balance responsible for excess body weight gain (energy gap) in young overweight children.

**Methods:**

Reported data on weight and height were used of 2190 Dutch children participating in the PIAMA birth cohort study. Accumulated body energy was estimated from the weight gain observed between age 2 and age 5-7. Energy gap was calculated as the difference in positive energy balance between children with and without overweight assuming an energy efficiency of 50%.

**Results:**

Ten percent of the children were overweight at the age of 5-7 years. For these children, median weight gain during 4-years follow-up was 13.3 kg, as compared to 8.5 kg in the group of children who had a normal weight at the end of the study. A daily energy gap of 289-320 kJ (69-77 kcal) was responsible for the excess weight gain or weight maintenance in the majority of the children who were overweight at the age of 5-7 years. The increase in daily energy requirement to maintain the 4.8 kilograms excess weight gain among overweight children at the end of the study was approximately 1371 kJ.

**Conclusions:**

An energy gap of about 289-320 kJ per day over a number of years can make the difference between normal weight and overweight in young children. Closing the energy gap in overweight children can be achieved by relatively small behavior changes. However, much more effort is required to lose the excess weight gained.

## Background

Worldwide, the prevalence of overweight continues to rise. Especially, the increase in overweight among children is of great concern. In 2000, 10% of all children aged 5-17 were overweight, a total of 155 million[1]. A further 22 million children below 5 years of age were also affected[1].

Overweight is the consequence of a long term positive energy balance where daily energy intake exceeds daily energy expenditure[2]. Therefore, overweight can theoretically be prevented by measures that restore or maintain energy balance. In 2003, Hill et al. introduced the term "energy gap", which was defined as the excess daily energy intake over daily energy expenditure that produces weight gain at the *population *level[3]. They reported that a deficit of 418 kJ (100 kcal) per day would be sufficient to prevent further weight gain in most of the adult population. According to this study, closing the energy gap in adults can be achieved by small behaviour changes, like by walking an extra mile each day or taking a few less bites of food at each meal. The findings reported by Hill et al. provide a quantitative target how much behaviour change is required to restore energy balance in the adult population.

As childhood overweight is increasing at a disturbing rate, it is important to have quantitative targets for the prevention of excess weight gain among children. Several studies attempted to quantify the energy gap in children[4-8]. Of those, three studies calculated the energy gap for *overweight *children particularly[6-8]. However, there were large differences in the estimated energy gap between those three studies, ranging from 418 - 586 kJ (100-140 kcal) per day[8] to 2837-4255 kJ (678-1017 kcal) per day[7]. Explanations for these differences are the use of other assumptions and different methods. Butte and Ellis[6] quantified the energy gap among Hispanic children living in the US based on 1-year changes in body composition and different assumptions on energetic efficiency, but did not take into account the energy necessary for growth. In 2006, Wang et al.[7] estimated the energy gap in US children, but used a cross-sectional design. They estimated excess weight from children aged 2-7 years in the period 1988-1994 and adolescents aged 12-17 years in the period 1999-2002 based on the normal weight distributions in 1988-1994.

A more accurate estimation of the energy gap in overweight children may come from prospective studies with a long-term follow up, which takes into account the limitations of previously conducted studies on this topic. Recently, Plachta-Danielzik et al. used a long-term longitudinal approach to calculate the energy gap in children aged 6-10[8]. So far, energy gap estimates for pre-school children are still lacking.

Aim of this longitudinal study was to quantify the energy gap responsible for the excess weight gain between age 2 and 5-7 years in overweight children of the PIAMA cohort. Hereby, it is taken into account that part of the positive energy balance is essential for normal growth.

## Methods

### Study design and study population

The study population consisted of young children who participated in the Dutch Prevention and Incidence of Asthma and Mite Allergy (PIAMA) birth cohort study. In this study, mothers were recruited from the general population during pregnancy (n = 4,146) and their children were born in 1996-1997. Nearly all women (97%) had a Western ethnic background. The study protocol was approved by the independent medical ethics committee of TNO and all parents gave written informed consent. A detailed description of the study design has been published previously[9].

Data were collected mainly by annual postal questionnaires, which included questions on the child's weight and height. The number of parents who completed the questionnaires ranged from 3,746 (90% of the baseline study population) when the children were 1 year old to 3,373 (81% of the baseline study population) when the children were 7 years old.

In the questionnaires parents were asked for the child's body weight (in kg) and height (in cm) the last time he or she was measured by a medical professional. When this measurement was not carried out in the last three months, parents were asked to weigh (without heavy clothes and shoes) and measure height of their child themselves. Also, date of the measurement and the person who carried out the measurements (parents or medical professional) were reported.

Body mass index (BMI) was calculated as body weight divided by height squared (kg/m^2^). Overweight was defined according to age and sex specific international BMI cut-off points for children from two till eighteen years, which are based on the adult overweight cut-off point of ≥ 25 kg/m^2^[10].

For this study, weight and height at the age of 2 years were used as baseline data, which were available for 2,489 children. As thirteen children were excluded because there were more than 8 weeks between the date of body weight measurement and the date of height measurement, BMI was calculated for 2,476 2-year-old children. Sex specific standard deviation scores of BMI (SDS or z-scores) for age were calculated using the reference growth curves of the Dutch Fourth Nation-wide growth study carried out in 1997[11].

To study changes in weight, height and BMI z-scores in these children, information at 5-7 years of age was used for comparison (end point). Finally, our study population consisted of 2,190 children for which BMI was available at the age of 2 and at the age of 5-7 years.

### Calculation and statistics

Changes in BMI-z scores, height and weight were calculated by subtracting the values at age 2 from the values at age 5-7. Based on overweight status at 2 and 5-7 years of age, children were divided into four groups. The first group consisted of children with a normal weight at both ages and is designated is this paper as "NN". The second group included children with a normal weight at the age of 2 and overweight at the age of 5-7 ("NO"). The third group consisted of children with overweight at both ages ("OO"). The fourth group included children with overweight at the age of 2 and normal weight at the age of 5-7 ("ON").

Differences in characteristics between the four groups were evaluated by one-way ANOVA for normally distributed variables. However the change in weight showed a skewed distribution and therefore were evaluated by Kruskal-Wallis test.

**Figure 1:**
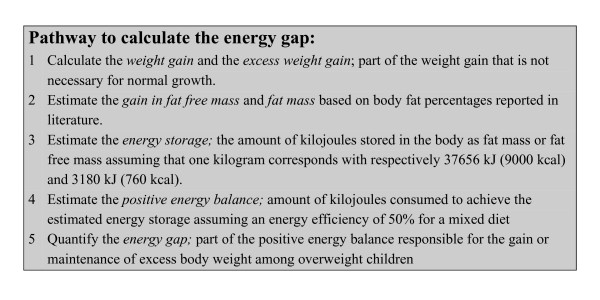
**Schematic overview of the different steps that have been conducted to calculate the energy gap among young overweight children**.

*Excess weight gain *was defined as part of the weight gain in children that is not necessary for healthy growth (figure 1). Excess weight gain was estimated 1) by subtracting the weight gain of children in the NN group from the weight gain of children in the NO group and 2) by subtracting the weight gain of children in the ON from the weight gain of children in the OO group.

Weight gain in children consists of a gain in fat mass and a gain in fat free mass. The absolute change in fat mass (kg) was estimated, based on the observed gain in body weight and body fat percentages for normal weight boys and girls at the age of 2 years and 5-7 years[12]. For example, a normal weight 2 year old boy has a body fat percentage of approximately 19.5% For overweight children we assumed a body fat percentage of 22.5% and 27.5% for respectively boys and girls[13].

The change in fat free mass was estimated by subtracting the estimated change in fat mass from the change in body weight. For example, a two year old boy weighs 14 kilograms, which is a normal weight for his age. His estimated fat mass is 2.7 kilograms (14 kg * 19.5%) and fat free mass is 11.3 kilograms (14 kg - 2.7 kg). At the age of six, the same boy has developed overweight and has a body weight of 27.5 kilograms. His body fat percentage is approximately 22.5%, his estimated fat mass is 6.2 kilograms (27.5 kg*22.5%) and fat free mass is 21.3 kilograms (27.5 kg - 6.2 kg). In four years this boy gained 3.5 kilograms fat mass (6.2 kg - 2.7 kg) and 10 kilogram fat free mass (21.3 kg - 11.3 kg).

*Energy storage *was defined as the amount of kilojoules stored in the body as fat mass or fat free mass. Assuming that one kilogram fat mass and one kilogram fat free mass correspond with respectively an energy storage of 37656 kJ (9000 kcal)[14] and 3180 kJ (760 kcal) (19% protein; 16.7 kJ (4 kcal) per gram protein)[6], the energy storage was calculated. Since, follow up time differs per child, we calculated the energy storage per day by dividing the energy storage by the exact follow up time in days.

The *positive energy balance *was defined as the amount of kilojoules consumed to achieve the calculated energy storage. Hereby, it should be taken into account that energy is not stored at 100% efficiency, owing in part to the metabolic costs of digesting and storing various ingested nutrients. We used the assumption made by Hill that energy derived from a mixed diet is stored with an efficiency of at least 50%[3]. This means that for every 50 kJ stored at most 100 kJ have been consumed extra.

The *energy gap *was defined as part of the positive energy balance responsible for the gain or maintenance of excess body weight. The energy gap has been quantified 1) by subtracting the positive energy balance of children who had normal weight both at baseline and at the end of the study period (NN) from the positive energy balance of children who became overweight (NO) and 2) by subtracting the positive energy balance of children who were overweight at baseline and reached a normal weight (ON) from the positive energy balance of children who remained overweight (OO). For the energy gap calculation, the median positive energy balance and the corresponding 90^th ^percentile was used.

Under the assumption of a linear accumulation of excess weight, the magnitude of the daily energy gap remains constant over time. Closing the energy gap will prevent further excess weight gain. However, to loose the excess weight previously obtained, a greater deficit on the energy balance is required[15]. This is due to the fact that excess weight leads to an increase in energy expenditure and therefore an increase energy requirement[16]. In children, the average additional daily energy expenditure associated with maintaining each extra kilogram of excess body weight assuming an average physical activity level has been estimated to be 142.8 kJ (34.14 kcal)[7]. We calculated the increase in energy requirement caused by the excess weight by multiplying the excess weight in overweight children by 142.8 kJ.

In addition, to explore the accuracy of the energy gap estimate, alternative assumptions regarding the body fat percentages and energy efficiencies were used to calculate the energy gap. Firstly, age and sex specific values for body fat percentages in overweight children were applied instead of assuming only sex specific values[17]. Secondly, fat and protein specific energy efficiencies of respectively 87% and 42% were used instead of assuming an energy efficiency of 50% for a mixed diet[18].

SAS software version 9.1 (SAS Institute, Inc., Cary, NC) was used for the calculations.

## Results

The percentages of children with normal weight and overweight at baseline and at the end of the study are presented in table 1. At the age of two, 7.4% of the children were overweight, which increased up to 10% when the children reached the age of 5-7 years. At both ages, more girls than boys were overweight. The majority of the children (85%) had a normal weight at both ages (NN). Five percent of the children were overweight at the age of two and achieved a normal weight at the age of 5-7 (ON). During the study, 7% of the children developed overweight (NO) and 3% of the children remained overweight (OO).

**Table 1:** Classification of 2190 children in normal weight and overweight at 2 and 5-7 years of age.

	Age (years)					
	
	2			5-7		
BMI-categories*	Total (n = 2190)	Girls (n = 1061)	Boys (n = 1129)	Total (n = 2190)	Girls (n = 1061)	Boys (n = 1129)
Normal weight (%)	92.6	91.1	94.1	90.0	87.2	92.7
Overweight (%)	7.4	8.9	6.0	10.0	12.5	7.4

BMI, body mass index.

* Classification is based on age and sex specific international BMI cut off points for children[10].

Table 2 presents BMI z-scores, height and weight at baseline and the changes in these variables between 2 and 5-7 years of age for the four groups based on BMI status. Children in the NO group already had a higher body weight (p < 0.0001) and BMI z-score (p < 0.0001) at the age of two compared with children in the NN group. Children in the ON group were shorter at the age of two than children in the other groups (p < 0.008). The median follow up time was 207.4 weeks and did not significantly differ between the four groups.

**Table 2:** BMI z-scores, height and weight at baseline (age of 2) and change during the study (until the age of 5-7) of 2190 children divided in four groups based on BMI status.

	BMI status at 2 and 5-7 years of age*								
	
	Normal/Normal (n = 1868)		Normal/Overweight (n = 160)		Overweight/Overweight (n = 59)		Overweight/Normal (n = 103)		P value***
					
	Mean	SD	Mean	SD	Mean	SD	Mean	SD	
*Age of 2:*									
BMI z-score	-0.25^bcd^	-0.91	0.43^acd^	0.79	2.00^ab^	0.50	1.85^ab^	0.43	<0.0001
Height (cm)	93.6^d^	5.2	94.0^d^	5.0	93.5^d^	6.0	91.2^abc^	5.0	<0.0001
Weight (kg)	13.9^bcd^	1.7	14.7^acd^	1.7	16.6^abd^	2.2	15.6^abc^	1.6	<0.0001
*Change:*									
BMI z-score	-0.19^bd^	0.95	1.12^acd^	0.92	-0.34^bd^	0.67	-1.50^abc^	0.55	<0.0001
Height (cm)	28.8^cd^	5.8	28.9^cd^	7.9	30.7^ab^	7.3	31.5^ab^	6.0	<0.0001
Weight (kg)**	8.5^bc^	7.0;10.3	13.4^ad^	10.5;16.0	13.1^ad^	10.5;15.2	8.7^bc^	7.5;10.0	<0.0001

BMI, body mass index. SD, standard deviation

^a ^Significantly different (p < 0.05) from group Normal/Normal.

^b ^Significantly different from group (p < 0.05) Normal/Overweight.

^c ^Significantly different from group (p < 0.05) Overweight/Overweight.

^d ^Significantly different from group (p < 0.05) Overweight/Normal.

*Classification is based on age and sex specific international BMI cut off points for children[10].

** Skewed distribution, median and 25^th ^and 75^th ^percentile presented.

***ANOVA for normally distributed variables, Kruskal Wallis for variables with a skewed distribution.

For children in the NN group, the median gain in body weight was 8.5 kilograms and the mean increase in height was 28.8 centimetres during the follow-up period. Furthermore, a small decrease in mean BMI z-score of -0.19 was observed in those children. Children in the OO and ON group grew faster in height than children in the NN and NO group. The excess weight gain was 4.9 kilograms (13.4 kg-8.5 kg) in children who developed overweight during the study (NO) and 4.4 kilograms (13.1 kg-8.7 kg) in children who remained overweight during the study (OO). For children in the OO and the ON group a decline in mean BMI z-score of respectively -0.34 and -1.50 was observed. Among children who developed overweight during the study (NO), the mean BMI z-score increased with 1.12.

Table 3 shows the daily gain in fat mass, fat free mass and body weight and also the daily energy storage and the positive energy balance assuming an energy efficiency of 50%. In the NN group, the weight gain consisted for 6% of an increase in fat mass (table 3). For children in the NO and OO group, the increases in fat mass expressed as part of the total weight gain were approximately five times higher and were respectively 34% and 28%. In the ON group, nearly the total body weight gain consisted of an increase in fat free mass.

**Table 3:** Daily increase in body weight, energy storage, positive energy balance and energy gap of 2190 children divided in four groups based on BMI status.

	BMI status at 2 and 5-7 years of age*							
	
	Normal/Normal (n = 1868)		Normal/Overweight (n = 160)		Overweight/Overweight (n = 59)		Overweight/Normal (n = 103)	
				
	Median	P90	Median	P90	Median	P90	Median	P90
Body weight gain (g/day)	6.2	8.1	9.4	12.4	9.0	12.5	6.1	7.4
Excess weight gain (g/day)			3.2		2.9			
Fat mass (%gained)	6.3	18.5	34.1	41.4	27.5	34.9	0	2.3
Fat free mass (%gained)	93.7	99.7	65.9	74.3	72.5	77.5	100	100
Energy storage (kJ/day)	32.7	52.5	137.3	196.7	112.8	187.4	19.5	27.2
Positive energy balance** (kJ/day)^3^	65.5	104.9	274.7	393.4	225.6	374.6	39.0	54.4
Energy gap (kJ/day)			209.2	288.5	186.6	320.2		

BMI, body mass index. P90, 90^th ^percentile.

* Classification is based on age and sex specific international BMI cut off points for children[10].

** Assuming an energetic efficiency of 50%[3].

**Figure 2:**
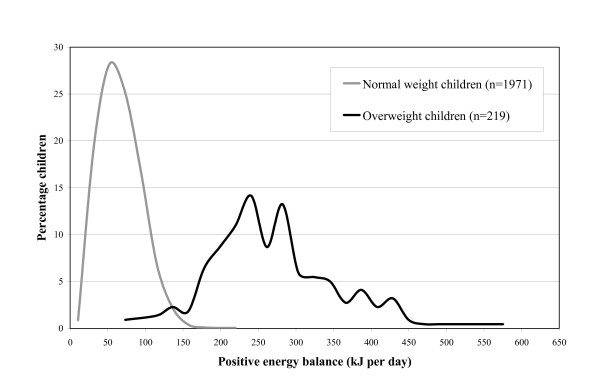
**Distribution of the estimated positive energy balance (kilojoules per day) during the study (median follow up 207.4 weeks) among normal weight and overweight children at the age of 5-7, The PIAMA study, n = 2190**.

For children in the NN group, the median energy storage was 32.7 kJ per day and the 90^th ^percentile was 52.5 kJ per day (Table 3). Children in the NO or OO group stored respectively 137.7 and 112.8 kJ per day. The lowest median energy storage was found among children of the ON group (19.5 kJ per day). As an energy efficiency of 50% was assumed, estimates of the positive energy balance are twice as high as the estimates of the energy storage. A visual presentation of the positive energy balance for 5-7 year old children with and without overweight separately is shown in figure 2. The median energy gap in the children who developed overweight (NO) was 209.2 kJ per day and the 90^th ^percentile was 288.5 kJ per day. Among children who remained overweight (OO), the median energy gap was 186.6 kJ per day and the 90^th ^percentile was 320.2 kJ per day.

The analyses were repeated using different assumptions. Using the macronutrient specific energy efficiencies resulted in an energy gap of about 188 kJ per day instead of 289-320 kJ per day. Applying age-specific values for body fat percentages in overweight children resulted in an energy gap of 230 kJ per day instead of 289-320 kJ per day.

We estimated the size of the daily energy gap responsible for the development of or maintenance of overweight in this population. This gap would have to be closed to avoid further excess weight gain. However, excess weight leads to an increase in energy expenditure and therefore an increase in energy requirement. In overweight children, the increase in daily energy requirement to maintain the 4.8 kilograms excess weight gain at the end of the study was approximately 1371 kJ per day. Therefore, a much larger deficit on the energy balance has to be created in order to restore normal weight

## Discussion

An energy gap of about 289-320 kJ (69-77 kcal) per day was responsible for the observed excess weight gain or maintenance in the majority of the children who where overweight at the end of the study. This amount of energy corresponds with for example one glass (approximately 200 ml) of soft drink per day. To burn 289-320 kJ children have to walk for about half an hour.

Strength of this study was its longitudinal character. The same group of children was used to calculate the change in body weight over a period of 4 years. Furthermore, in this study the energy necessary for normal growth was taken into account in estimating the energy gap among overweight children.

Although loss-to-follow-up and non-response on specific questionnaires were relatively low in the PIAMA study, a considerable proportion of the children had missing values on BMI. In the first year of life, when children are regularly weighed and measured in under-five clinics, response on the weight and height questions was high, but it decreased in the second year of life and even further in later years. In some cases parents reported only weight or height, but not both measurements, or they failed to report the dates of the two measurements, so that the age of the child at the time of measurement could not be calculated. Children with missing BMI data at age 2 more often had a lower educated mother than children with known BMI data at age 2 (data not shown). As obesity is more common among people with lower SES, we may have underestimated the percentages of overweight and obesity at age 2 However, there were no differences in BMI z-scores at age 1 between these groups. Furthermore, mothers of children with known BMI at age 2 and missing BMI data at age 5-7 were also more often lower educated than mothers of children with known BMI data at age 2 and age 5-7. However, there were no differences in BMI at age 2 between those groups. In addition, we observed no difference in weight gain during the study between children grouped by educational level of their mother. Therefore, we think that missing values in BMI has not materially influenced our energy gap estimates.

In this study, parental reported data on height and weight was used to estimate the energy gap. A study among 4 year old children who participated in the PIAMA cohort concluded that overweight prevalence rates are underestimated when based on reported weight and height[19]. Parents of children with a high BMI tend to underreport, whereas parents of children with a low BMI tend to over report their child's body weight, both with 0.5 kilograms on average. Therefore, the self reported data on weight may have resulted in an underestimation of weight gain of at most one kilogram in children who became overweight. At an energy efficiency of 50%, the calculated energy gap may therefore have been underestimated by at most 52 kJ per day in overweight children.

Fat mass percentages can differ substantially between normal weight individuals as well as overweight individuals[20]. Unfortunately, information about the body composition of the children was not available in this study. Therefore, assumptions about body fat percentages were made based on literature [12,13]. We also had to make assumptions about the energy efficiency. A commonly used assumption that a mixed diet is stored with an efficiency of 50% was used to facilitate the comparison with other studies that quantified the energy gap.

In a sensitivity analyses, we checked the validity of these assumptions. Firstly, we applied age and sex specific body fat percentages for normal weight and overweight children specific[17]. Secondly, we used protein and fat specific assumptions regarding energy efficiency. Both adjustments resulted in a lower estimate of the energy gap. Therefore, it seems that our energy gap estimate of 289-320 kJ per day is more likely to be an overestimate than an underestimate of the actual energy gap.

This study reported an excess weight gain of about 1.1 kilograms per year and a daily energy gap of 289-320 kJ among the majority of the Dutch overweight children aged 5-7. This finding is generally in line with the results recently published by Plachta-Danielzik et al. who estimated the energy gap in older children aged 6-10 years[8]. They reported a yearly excess weight gain of 2.5 kilograms. The higher energy gap reported (527 kJ (126 kcal) per day) in children who developed overweight during the study period can largely be explained by the higher excess weight gain. They calculated excess weight gain from changes in fat mass and fat free mass z-scores which were based on measured body composition. As mentioned before we had no measured data on fat mass and fat free mass. However, when applying their approach using body weight z-scores, resulted in an only slightly lower median excess body weight gain (1 kg/year vs 1.2 kg per year) among NO children and subsequently a slightly lower energy gap. The similarity between the findings shows that our estimate of the energy gap is rather robust.

Our estimation of the energy gap is much lower than the other two studies that estimated the energy gap among overweight children. Butte and Ellis calculated an energy gap of 1431-2100 kJ (342-502 kcal) per day among US overweight children aged 5-19, at an energy efficiency of 50%[6]. This larger energy gap can be explained by the fact that the energy required for healthy growth among children was not taken into account and by the higher excess weight gain of about 2.7 kilograms per year among US overweight children.

Wang et al.[7] reported the highest energy gap of 2837-4255 kJ (678-1017 kcal) per day among overweight US children aged 12-17. The yearly excess weight gain was similar to that reported by Butte and Ellis and therefore can not explain the higher energy gap. However, Wang et al. added the increase in daily energy requirement to maintain the previously gained excess body weight to their daily energy gap estimate, whereas we (and the other authors) estimated the daily energy gap that would have to be closed to avoid further excess weight gain. As, over a study period of 10 years, excess weight gain increased up to 26.5 kilograms, this has substantially influenced the magnitude of the energy gap estimated by Wang et al. In our study, the increase in daily energy requirement among overweight children at the end of the study was 1371 kJ due to a body weight gain of 4.8 kilograms. Further excess weight gain among overweight children can be prevented by closing the energy gap. However, much more effort is required to lose the excess weight gained.

From the above it becomes clear that the different energy gaps reported in the literature might result from differences in the methodological approaches used to calculate the energy gap. However, it cannot be excluded that the observed differences could also in part reflect real life-course specific differences in energy balance. This could imply that specific recommendations for specific life periods are needed. Therefore it's worthwhile to study this issue further.

Interestingly, we found a relatively high remission rate in overweight in children between age 2 and age 5-7 (64%). This should also be taken into account in future recommendations on the prevention of childhood overweight.

Important to notice is that the estimated energy gap is a mean value for young Dutch children with overweight. Translation to individuals can not directly be made. For example, genetic variation may results in differences in energy efficiency between persons and therefore probably also in energy gap between individuals[21]. Furthermore, the estimated energy gap of 289-320 kJ is not applicable to more severe forms of overweight. For example, among children who became obese during the study, the calculated excess on the energy balance was 377 kJ (90 kcal) per day (data not shown).

## Conclusions

An energy gap of about 289-320 kJ (69-77 kcal) per day over a number of years can make the difference between normal weight and overweight in young children. Closing the energy gap in overweight children can be achieved by relatively small changes in diet or physical activity, like consuming 1 glass of soft drink per day less or increase walking for about half an hour per day. However, larger changes in energy balance are required to lose the excess weight gained.

## Abbreviations

NN: normal weight at the age of 2 and at the age of 5-7; NO: normal weight at the age of 2 and overweight at the age of 5-7; ON: overweight at the age of 2 and normal weight at the age of 5-7; OO: overweight at the age of 2 and at the age of 5-7; P90: 90th percentile; PIAMA: Prevention and Incidence of Asthma and Mite Allergy; BMI: body mass index.

## Competing interests

The authors declare that they have no competing interests.

## Authors' contributions

SWB was responsible for the statistical analysis, interpretation of the results and the draft of the manuscript. JMAB contributed to the conception and design of the current study, interpretation of the results and revision of the manuscript. SS contributed to the statistical analysis and critical revision of the manuscript. AHW contributed to the coordination of data acquisition and helped in conception and design of the current study, interpretation of the results and revision of the manuscript. JCJ, HAS and BB are responsible for the conception, design and coordination of the PIAMA project as a whole and were involved in critically revising the manuscript. All authors read and approved the final manuscript.

## Pre-publication history

The pre-publication history for this paper can be accessed here:

http://www.biomedcentral.com/1471-2458/11/326/prepub
